# Development of a Solid-Phase Extraction (SPE) Cartridge Based on Chitosan-Metal Oxide Nanoparticles (Ch-MO NPs) for Extraction of Pesticides from Water and Determination by HPLC

**DOI:** 10.1155/2018/3640691

**Published:** 2018-10-02

**Authors:** Mohamed E. I. Badawy, Mahmoud A. M. El-Nouby, Abd El-Salam M. Marei

**Affiliations:** Department of Pesticide Chemistry and Technology, Faculty of Agriculture, Alexandria University, El-Shatby, Alexandria 21545, Egypt

## Abstract

The present study aims to prepare two new types of chitosan-metal oxide nanoparticles (Ch-MO NPs), namely, chitosan-copper oxide nanoparticles (Ch-CuO NPs) and chitosan-zinc oxide nanoparticles (Ch-ZnO NPs), using sol-gel precipitation mechanism, and test them new as adsorbent materials for extraction and clean-up of different pesticides from water. The design of core-shell was implemented by metal oxide core with chitosan as a hard shell after crosslinking mechanism by glutaraldehyde and then epichlorohydrin. The characterizations of the prepared nanoparticles were investigated using Fourier transform infrared spectrometry (FT-IR), zeta potential, scanning electron microscopy (SEM), transmission electron microscope (TEM), and X-ray diffraction (XRD). FT-IR confirmed the interaction between chitosan, metal oxide, and crosslinking mechanism. SEM and TEM explained that the nanoparticles have a spherical morphology and nanosize of 93.74 and 97.95 nm for Ch-CuO NPs and Ch-ZnO NPs, respectively. Factorial experimental design was applied to study the effect of pH, concentration of pesticide, agitation time, and temperature on the efficiency of adsorption of pesticides from water samples. The results indicated that optimum conditions were pH of 7, temperature of 25°C, and agitation time of 25 min. The SPE cartridges were then packed with Ch-MO NPs, and seven pesticides of abamectin, diazinon, fenamiphos, imidacloprid, lambda-cyhalothrin, methomyl, and thiophanate-methyl were extracted from water samples and determined by HPLC. The extraction efficiency of Ch-ZnO NPs was higher than Ch-CuO NPs, but both removed a larger amount of most of tested pesticides than the standard ODS cartridge (C18). The results showed that this method achieves rapid and simple extraction in small quantities of adsorbents (Ch-MO NPs) and solvents. In addition, the method is highly sensitive to pesticides and has a high recovery rate.

## 1. Introduction

Pesticides are widely used in agricultural production to prevent or control pests, diseases, weeds, and other plant pathogens in an effort to minimize or eliminate yield losses and maintain high quality of products [1, 2]. Widespread uses of pesticides with all groups such as organochlorines, organophosphorus, carbamates, pyrethroids, and neonicotinoids have resulted in extensive contamination of water, atmosphere, and soil as well as agricultural products which eventually lead to food safety issues [3]. Water contamination with pesticides is considered a serious problem and may pose a risk to human health such as acute neurological toxicity, neurodevelopmental impairment, cancer, allergies, neurological disorders, and reproductive disorders [2, 4–6].

Different analytical techniques have been used for sample preparation and clean-up with differentiation of sensitivity and selectivity [7], which include liquid-liquid extraction (LLE) [8], solid-phase extraction (SPE) [9], solid-phase microextraction (SPME) [10], dispersible solid-phase extraction (d-SPE), headspace solid-phase extraction [11], and stir bar sorptive extraction (SBSE) [12]. SPE was introduced in the early 1970s to avoid and minimize the disadvantage of LLE technique. The SPE is a superior extraction and clean-up method that uses a solid phase and a liquid phase to separate the analyte from the sample without impurities before analysis by dint of speed, less usage of organic solvent, low cost, and ability to obtain a higher preconcentration factor [13, 14]. Recently, advanced materials for SPE extraction have been investigated with separation by liquid chromatography and ultraviolet absorption detection (HPLC/UV) [15–17].

Some of the most common sorbents in SPE are generally similar to those in column liquid chromatography such as the primary secondary amine (PSA), octadecyl-siloxane (C_18_), graphitized carbon black (GCB), alumina, and florisil. PSA is normally used in the d-SPE to remove interferences, such as free fatty acids, sugars, and other nonpolar compounds from the sample. However, the most commercial stationary phase used in SPE is octadecyl-siloxane (C_18_) used in the reversed phase to extract the nonpolar compounds like pesticides [18, 19].

Recently, the biopolymer materials have been shown to be of low cost and good efficiency in removal of various contaminants from aqueous media. Among these biopolymers, chitosan (poly-*β*-(1→4)-2-amino-2-deoxy-D-glucose) [20, 21] has been considered to be one of the most promising and applicable materials in adsorption applications [22]. The existence of the two functional groups of hydroxyl (-OH) and amino (-NH_2_) in its molecular structure contributes to many possible adsorptions and gives highly powerful removal capacity of dyes, metal ions, phenols, pharmaceuticals drugs, and other pollutants including the pesticides from environment and wastewater [23].

Metal oxide particles have been used in many functions [24] in various polymeric materials to improve the permanence of the polymeric products [25]. In addition, the nanoparticles of these products could increase the stiffness, toughness, and service life of polymeric materials [26]. The chitosan-metal oxide complexes in nanostructure form have been extensively modified to improve the adsorption capacity of chitosan molecule because of their limited size and a high density in their corner or edge surface sites [6]. Dehaghi and coauthors synthesized the chitosan-ZnO nanoparticles (Ch-ZnO NPs) for adsorption applications in the removal of pesticide pollutants [25]. They found that the 0.5 g of the Ch-ZnO NPs, in room temperature and pH 7, removed 99% of permethrin insecticide solution (0.1 mg/L). Copper-coated chitosan nanocomposite (Ch-Cu) was prepared and used for adsorption of parathion and methyl parathion insecticide in the batch mode. The maximum adsorption capacity of malathion was found to be 322.6 mg/g at an optimum pH of 2.0. The adsorbent was found to remove malathion completely from the spiked concentration of 2 mg/L in one min in the agricultural run-off samples [24].

Chemical modification promotes crosslinking of the polymer chains. This process consists of joining polymer chains with the help of high reactivity chemicals called crosslinking agents, generating polymer networks. This modification type is only possible by the presence of functional groups of high reactivity in the structure of these polymers. Most notably, glutaraldehyde and epichlorohydrin as crosslinking agents considerably improve the mechanical strength, the hardness of the chitosan particles, and the chemical stability in acidic media [27, 28]. Epichlorohydrin was selected as a convenient base catalyzed crosslinking agent. An advantage of epichlorohydrin is that it does not eliminate the cationic amine function of chitosan, but it reacts with hydroxyl groups in chitosan. Glutaraldehyde has been used more frequently since it is less expensive, nontoxic, and highly soluble in aqueous solution. It is a dialdehyde whose aldehydic groups are highly reactive and can form covalent bonds with functional groups such as primary amine by Schiff base suggesting that the conjugated aldehyde moieties in the polymers yield more stable reaction products [29–31].

In the current study, new chitosan-metal oxide nanoparticles (Ch-MO NPs) including chitosan-CuO nanoparticles (Ch-CuO NPs) and chitosan-ZnO nanoparticles (Ch-ZnO NPs) were prepared through the crosslinking mechanism by glutaraldehyde and then epichlorohydrin. The nanoparticles were used as a stationary phase in the preparation of SPE cartridge. The SPE cartridges were used in extraction and clean-up of pesticides from water samples. The efficiency of the prepared cartridge of adsorption or retention of the different pesticides including abamectin, diazinon, fenamiphos, imidacloprid, lambda-cyhalothrin, methomyl, and thiophanate methyl was tested at three concentrations of each pesticide. The targeted pesticides are known to have been extensively used in agriculture in Egypt. The pesticide residues were determined by HPLC system. This protocol addresses the detection of trace amounts of these pesticides in water and optimizes the conditions for SPE technique compared with the commercial SPE of Supelco Sigma product.

## 2. Materials and Methods

### 2.1. Chemicals

Low molecular weight of acid-soluble chitosan (3.60 × 10^5^ Da and 88% degree of deacetylation), glutaraldehyde (50%), epichlorohydrin (99%), toluene, dimethylformamide, and ethyl acetate were purchased from Sigma-Aldrich Co (St. Louis, Missouri, USA). HPLC-grade of acetonitrile, methanol, and water were purchased from Carlo-Erba Reagents SAS, Co (Chaussee du Vexin, 27100 Val-de-Reuil, France). Zinc oxide (ZnO), red copper (I) oxide (Cu_2_O), acetic acid, nitric acid, and sodium hydroxide were purchased from El-Gomhoria for pharmaceutical and chemicals Co (Adeb Ishak St, Manshia, Alexandria, Egypt) and used without further purification.

### 2.2. Technical Pesticides

Technical grade of abamectin (96% purity) was purchased from Merck and Co., Inc., (Kenilworth, New Jersey, USA). Chlorpyrifos methyl (97%) was purchased from Dow Chemical Co., (Midland, Michigan, USA). Diazinon (90%) was purchased from Syngenta International AG Co, (Schwarzwaldallee 215, 4002 Basel, Switzerland). Fenamiphos (90%) was purchased from Miles Inc, Co, (8400 Hawthorn Road, Stilwell, Kansas City, USA). Imidacloprid (96%) was purchased from Bayer AG Co (51368 Leverkusen, Germany). Lambda-cyhalothrin (97%) was purchased from Syngenta International AG Co (Schwarzwaldallee 215, 4002 Basel, Switzerland). Methomyl (98%) was purchased from E.I. du Pont de Nemours and Co (Wilmington, Delaware 19805, USA) and thiophanate-methyl (94%) was purchased from Pennwalt Ltd, Co, (D-221, M.I.D.C, T.T.C. Industrial Area, Thane Belapur Road, Nerul, Navi Mumbai, Maharashtra, India). The chemical structures of these pesticides are shown in Figure S1.

### 2.3. Instruments and Equipment

High-Performance Liquid Chromatography (HPLC) Agilent technology infinity 1260 (Germany) equipped with an Agilent variable wavelength ultraviolet detector (VWD) was used. The system consists of a quaternary gradient solvent pump to control the flow rate of the mobile phase and an autosampler for automatic injection with a 100 *μ*L sample loop, a vacuum degasser, and a column oven (5-80°C). Separation was performed on ZORBAX Eclipse Plus C18 analytical column (250 × 4.6 mm id, 5 *μ*m particle size). Data were managed using HP Chemstation software. Perkin Elmer FT-IR Spectrophotometer L160000A with detector LiTaO_3_, PerkinElmer, Inc, (Waltham, Massachusetts, USA); Malvern Zeta-Nano-sizer, using Laser Doppler Micro-Electrophoresis Malvern instrument Ltd Co (Enigma Business Park, Grove wood Road, Malvern WR14 1XZ, UK); UV-visible Spectrophotometer Alpha 1502 (Laxco, Inc., Bothell, WA 98021, USA); scanning electron microscope (SEM) JSM5300, JEOL Ltd, (Akishima, Tokyo, Japan); transmission electron microscope (TEM) JEOL JEM-1400 (USA); Bruker's X-ray diffraction (XRD, USA); ultrasonic homogenizer HD 2070 with HF generator (GM 2070), ultrasonic converter UW 2070, booster horn (SH 213 G), and probe microtip MS 73, Ø 3 mm, BANDELIN electronic GmbH & Co. (KG. Heinrichstraße, Berlin, Germany); hotplate with magnetic stirrer, IKA-Werke GmbH & Co (Breisgau-Hochschwarzwald, Germany); oven, Heraeus Co (KG-Hanau, Germany); and electric balances three and four digits, BL-410SLCD, Setra systems Inc, (59 Swanson Rd, Boxborough, MA 01719, USA) were used.

### 2.4. Preparation of Chitosan-Metal Oxide Nanoparticles (Ch-MO NPs)

Ch-MO NPs including chitosan-copper oxide (Ch-CuO) and chitosan-zinc oxide (Ch-ZnO) nanoparticles were prepared according to the method of Dehaghi and others with minor modifications [25]. A weight (4 g) of chitosan was dissolved in 100 mL aqueous acetic acid solution (1%, v/v) and stirred for 2 h using magnetic stirrer (solution A). The desired amount of metal oxide (1 mol metal ions per 1 mol amino group of chitosan) was added to the solution. In the case of Ch-Cu complex, Cu_2_O (7.09 g) was dissolved in 20 mL diluted nitric acid (2%, v/v) (solution B); however, in the case of Ch-Zn complex, ZnO (8 g) was dissolved in 10 mL concentrated nitric acid (solution C). Solution B or C was added dropwise to the solution A using a syringe under continuous stirring for 2 h until the metal ions conjugated with a chitosan polymer. After that, 12 mL of glutaraldehyde (50%, v/v), as a first crosslinking agent, was added dropwise to the mixture under stirring, followed by addition of 8 mL epichlorohydrin (99%) as a second crosslinking agent, under continuous stirring. The pH was adjusted to 10 by NaOH (1N) dropwise by syringe under stirring. The reaction mixture was then sonicated for 15 min at a sonication power of 10 kHz and pulses or cycles (9 cycle /sec). Finally, the solution was stored in a water bath at 60°C for 3 h until precipitation. The precipitate was filtered, washed with distilled water, and dried at 70°C for 3 h.

### 2.5. Characterizations of Ch-MO NPs

#### 2.5.1. Scanning Electron Microscope (SEM)

The samples of Ch-MO NPs were investigated using a JEOL SEM with a magnification of 20000x and acceleration voltage of 19 kV. The dry particles were suspended in ethyl alcohol by sonication in dismantling the assembled particles. After that, the particles were mounted on metal stubs with double-sided tape, sputtered with gold, and viewed in an SEM.

#### 2.5.2. Transmission Electron Microscope (TEM)

TEM observation was performed on a JEOL JEM-1400 electron microscope (USA) at accelerating voltage of 120 kV. Specimens for TEM measurements were prepared by depositing a drop of colloid solution on a 400 mesh copper grid coated by an amorphous carbon film and evaporating the solvent in air at room temperature.

#### 2.5.3. X-Ray Diffraction (XRD)

X-ray diffractograms on powder samples were obtained using a Bruker's X-ray diffraction (USA) with Cu tube radiation (*k* = 1.54184 Å), a graphite monochromator and Lynxeye detector at 30 kV, and a current of 10 mA. The diffractometer was controlled and operated by a PC computer with the DIFFRAC.SUITE™ software package. Measurements were taken over an angular range of 0.99° ≤ 2*θ* ≤ 89.99° with a scanning step of 0.05 and a fixed counting time of 10 s. Divergence, scattered, and receiving radiation slits were 1°, 1°, and 0.2 mm, respectively.

#### 2.5.4. Zeta Potential

The surface charge of Ch-MO NPs was investigated by a Malvern Zeta-Nano-sizer instrument. The fixed weight (0.1gm) of the prepared particles was suspended in glycerol (50%) in isopropanol (v/v) and then they were sonicated for 30 min. The suspension was transferred to zeta potential cell [32].

#### 2.5.5. FT-IR Spectroscopy

The functional groups of Ch-MO NPs was analyzed by FT-IR spectroscopy with KBr discs (5 mg of Ch-MO NPs and 100 mg KBr pellets), in the range from 4000 to 400 cm^−1^, with a resolution of 4.0 cm^−1^ on a Perkin Elmer 1600 FT-IR Spectrophotometer (USA) [20].

### 2.6. Kinetic Study

The preliminary study was conducted to investigate the influence of some factors (pH of the solution, temperature, and agitation time) on the adsorption efficiency of imidacloprid (as a pesticide example) on Ch-CuO NPs using full factorial design in MINITAB® software v17.1.0, 2002 (Minitab Inc, Co., Pine Hall Rd, State College, PA 16801-3008, USA). The three factors were tested at three levels including low level, high level, and medium level, coded as -1, +1, and 0, respectively. The minimum number of experimental runs that have to be carried out for two levels with three factors design is 2^3^ = 8 runs plus 1 run at a center point. The experiments were carried out using 100 mg of each type of nanoparticles suspended in 25 mL of imidacloprid solution (25 mg/L) at 10, 25, and 40°C, pH 5, 7, and 9, and different agitation times (10, 25 and 40 min) with shaking at 150 rpm. The blank samples were added and placed in the same shaker to avoid loss of evaporation of pesticide or solvent. After each time with different experiments, the eluent was determined by HPLC [2, 25, 33].

### 2.7. Solid-Phase Extraction (SPE) of Different Pesticides by Ch-MO NPs

**Figure 1:**
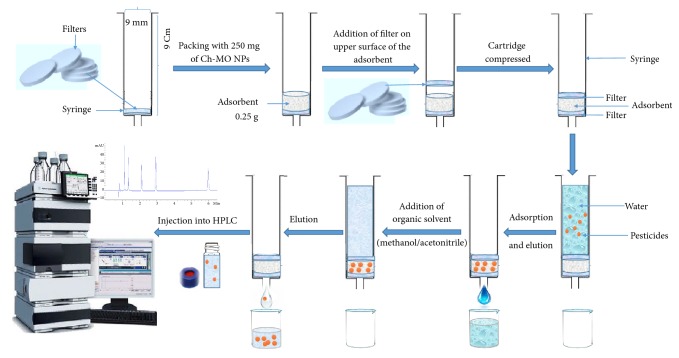
A schematic diagram shows extraction and clean-up of pesticides using SPE cartridge packed with Ch-MO NPs (Ch-CuO NPs and Ch-ZnO NPs). This figure is reproduced from Badawy et al. (2018) (under the Creative Commons Attribution License/public domain).

The prepared nanoparticles were studied as solid matrix materials in SPE cartridge. The SPE cartridge was performed using a plastic syringe column of 0.9 cm diameter and 9 cm in length (Figure 1). The column was filled up without gaps by compressing a frit on the bottom and then adding 0.25 g of each Ch-MO NPs and stopcock frit on the upper [34]. We compared these cartridges with the ODS (C18, Supelco) cartridge as it is the most common material used in extraction and clean-up of pesticide residues. Three different concentrations (10, 50, and 100 mg/L) of each pesticide (abamectin, diazinon, fenamiphos, imidacloprid, lambda-cyhalothrin, methomyl, and thiophanate-methyl) were prepared by dissolving the tested pesticide in a minimum volume of methanol and then completed to the final volume of 20 mL with water. The prepared solutions were allowed to pass through the SPE cartridge. After that, the adsorbed amount of each pesticide was eluted by 5 mL of acetonitrile/methanol (1:1, v/v).

### 2.8. HPLC Analysis

The water phase (effluent) and organic phase (eluent) were collected from SPE cartridge and injected into HPLC. The summary of the optimum conditions for chromatographic analysis of each pesticides is presented in Table S1. For analysis calibration, five standard solutions of each pesticide were prepared by dissolving weighed amount in the mobile phase used for each pesticide, and different quantities (0.0125-0.15 *μ*g/mL) were injected into HPLC. Calibration curves were constructed by plotting the peak areas of compound against the amount injected in *μ*g. Regression analysis of the data (n = 5) for each calibration curve gave the values of slope, along with the intercept and correlation coefficient. Calibration curves were used for the quantification of the pesticides in water samples. The limit of detection (LOD) and limit of quantification (LOQ) for each pesticide were calculated. The LOD is the lowest concentration of the analyte in a sample that can still be detected by the analytical method but should not be quantified as an appropriate value. However, the LOQ is the lowest concentration of the sample that can still be quantitatively detected with acceptable precision and accuracy [35]. LOD was defined as 3*σ*/S and LOQ was defined as 10*σ*/S, where *σ* is the standard deviation and S is the slope of the calibration curve [36].

### 2.9. Statistical Analysis

The statistical analysis was performed using the SPSS 25.0 software (Statistical Package for Social Sciences, USA). Analysis of variance (ANOVA) of the data was conducted, and means property values were separated by Student-Newman-Keuls (SNK) test. Differences were considered significant at p ≤ 0.05. The statistical analysis of adsorption kinetics was investigated by full factorial design using a MINITAB® software v17.1.0, 2002 (Minitab Inc, Co., Pine Hall Rd, State College, PA 16801-3008, USA).

## 3. Results and Discussion

### 3.1. Preparation of Ch-MO NPs

The Ch-MO NPs were synthesized through combining the sol-gel precipitation and crosslinking mechanism [27] as illustrated in Figure S2. Monodispersed metal oxide particles were coated by chitosan as the uniform of core or shell layer. They were then sequentially crosslinked with glutaraldehyde and epichlorohydrin. Firstly, glutaraldehyde forms the hard-spherical shape of particles through reaction with the amino groups of chitosan. In the second stage, the epichlorohydrin reacted with the hydroxyl groups to give more hardness for particles and reduce the hydrophilicity of chitosan. The final product was precipitated by aqueous solution of NaOH (1N). The yields were 85.29% and 91.67% for Ch-CuO NPs and Ch-ZnO NPs, respectively, with a yellowish and dark yellowish color, respectively (Table 1).

Many research articles prepared and characterized polymer-supported metals and metal oxide nanoparticles including chitosan-ZnO and chitosan-CuO, and some of which suggested the previous mechanism of the particle formation [26, 37]. For example, Shrifian-Esfahni et al. prepared and characterized Fe_3_O_4_/chitosan core-shell and the mechanism investigated hydrogen-bonding formation. In addition, the authors indicated the unbonded hydroxyl groups with partial positive charges surrounding nanoparticle [37]. Therefore, we completed this reaction in our study by crosslinking agent to cover the reactive functional groups (amino and hydroxyl). Recently, we prepared chitosan‐siloxane magnetic nanoparticles from Fe_3_O_4_ functionalized by siloxane derivatives followed by coating with chitosan through a crosslinking mechanism using glutaraldehyde and epichlorohydrin [34].

### 3.2. Characterizations of Ch-MO NPs

#### 3.2.1. Scanning Electron Microscope (SEM)

**Figure 2:**
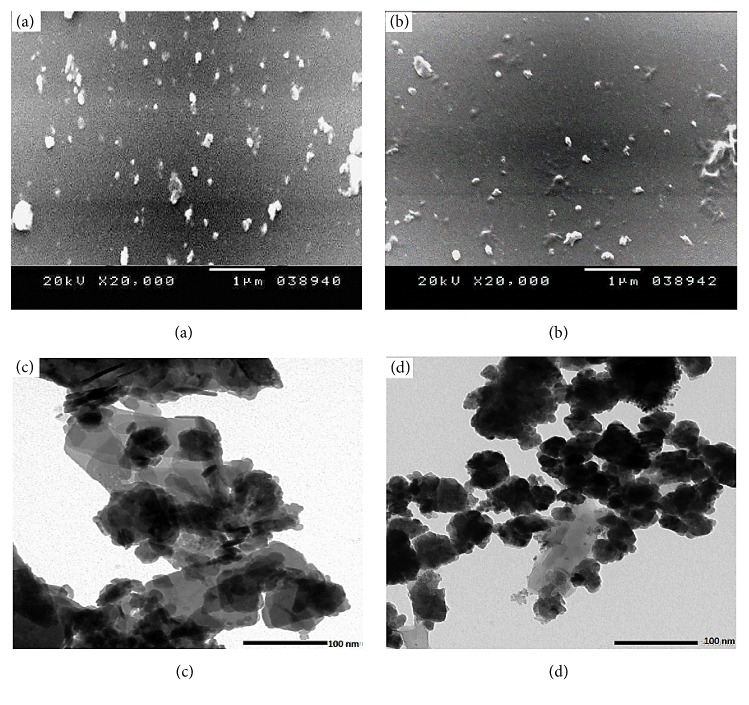
Electron microscopy images of Ch-MO NPs.** (a), (b)** The SEM of Ch-CuO NPs and Ch-ZnO NPs, respectively.** (c), (d)** The TEM of Ch-CuO NPs and Ch-ZnO NPs, respectively. Scale bar for SEM measurements was 1 *μ*m and magnification x20000 at 20 Kv. Scale bar for TEM measurements was 100 nm and magnification x40000 at 20 Kv.

The SEM was used to investigate the surface morphology and particle size of Ch-CuO NPs and Ch-ZnO NPs as shown in Figures 2(a) and 2(b), respectively. The particles in nanocomposites were found with almost spherical morphology with aggregations of the nanoparticles. Nanoparticles were measured with an average size of 93.74 and 97.95 nm for Ch-CuO NPs and Ch-ZnO NPs, respectively (Table 1). Dehaghi and coauthors prepared Ch-ZnO NPs without crosslinking reaction and they found that the particles size was in a arrange of 58 nm [25]. However, Manikanndan and others prepared the Ch-Cu complex without crosslinking reactions with an average size ranging from 20 to 30 nm [38]. Gouda and Hebeish loaded CuO NPs into chitosan by using drops of H_2_O_2_ (30%) and then stirring with a high-speed homogenizer at 10000 rpm for 30 min. The corresponding CuO/chitosan nanocomposite formed was characterized by using transmission electron microscope (TEM) images and they presented a very homogeneous morphology with a quite uniform particle size distribution and a rather spherical shape [39]. The particle size diameters obtained were 10 nm for chitosan nanoparticle and 20 nm for CuO/chitosan nanocomposite.

#### 3.2.2. Transmission Electron Microscope (TEM)

TEM photographs of Ch-CuO NPs and Ch-ZnO NPs are presented in Figures 2(c) and 2(d), respectively. It is evident that the particles are formed with average sizes ranging from 75 to 100 nm. In addition, the nanoparticles of both products showed high agglomeration of smaller size nanoparticles and their surface was rough and porous because metal oxide particles were wrapped by chitosan matrix.

#### 3.2.3. X-Ray Powder Diffraction (XRD)

**Figure 3:**
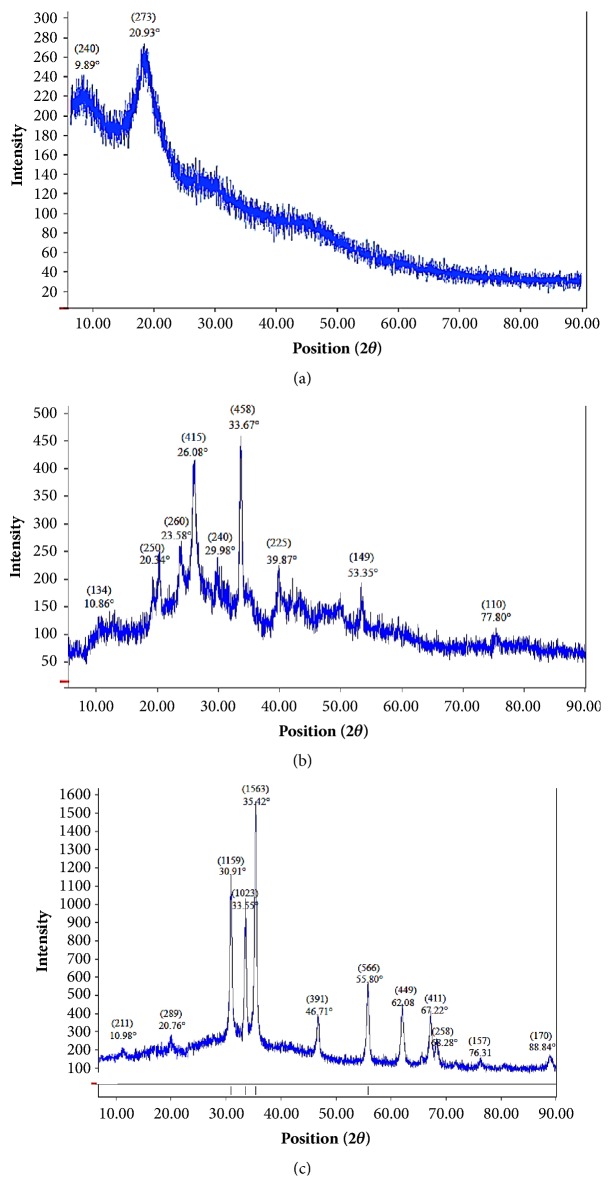
X-ray diffraction (XRD) patterns of chitosan (a), Ch-CuO NPs (b), and Ch-ZnO NPs (c).

The X-ray diffraction patterns of Ch-MO NPs are shown in Figure 3.  Figure 3(a) shows the characteristic peaks at 2*θ* ~ 10° and 2*θ* ~ 20°, due to inter- and intramolecular hydrogen bonds in chitosan molecule [40, 41]. However, these two peaks are very weak in the spectra of Ch-CuO NPs and Ch-ZnO NPs (Figures 3(b) and 3(c), respectively), which suggest a low crystallinity and an amorphous nature of the products. The weak peaks reflect great disarray in chain alignment of chitosan with the production of new peaks identifying zinc oxide and copper oxide. The X-ray diffraction patterns of Ch-CuO NPs (Figure 3(b)) demonstrated diffraction angles of 23.58°, 26.08°, 29.98°,33.67°,39.87°, 53.35°, and 77.80°, which correspond to the characteristic face centered CuO core with counts index (260), (415), (240), (458), (255), (149), and (110), respectively [42, 43]. The diffraction angles observed at 10.86° and 20.34° corresponding to count indexes (134) and (250), respectively, refer to the chitosan shell. The main peaks of Ch-ZnO NPs (Figure 3(c)) were at 2*θ* = 30.91°, 33.55°, 35.42°, 46.71°, 55.80°, 62.08°, 67.22°, and 68.28°, which correspond to the (1159), (1023), (1563), (391), (566), (449), (411), and (258) crystal planes, respectively. These peaks are consistent with the database in Joint Committee on Powder Diffraction Standards for ZnO (JCPDS file, PDF No. 36-1451) [44]. In addition, two smaller peaks at 2*θ* = 76.31° and 88.84° corresponding to the count (157) and (170), respectively, were also observed. The diffraction angles observed at 10.98° and 20.76° corresponding to count indexes (211) and (289), respectively, refer to the chitosan shell.

#### 3.2.4. Zeta Potential

Zeta potential is the surface charge value and it is a key indicator of the stability of colloidal dispersions. The magnitude of the zeta potential indicates the degree of electrostatic repulsion between charged particles in a dispersion. For molecules and particles that are small enough, a high zeta potential will confer stability; i.e., the solution or dispersion will resist aggregation [32, 45]. In the present study, the values were +0.516 mV for Ch-CuO NPs and +0.086 mV for Ch-ZnO NPs (Table 1 and Figure S3), indicating a rapid coagulation or flocculation of particles in suspension at pH 7 and 25°C. It can be noted that the nanoparticles of Ch-CuO NPs have a higher charge (≈ 5-fold) than Ch-ZnO NPs. The positive charge of zeta potential values obtained refers to the surface charge of the particles. The previous study reported that the Ch-Cu complex has a negative charge (-29 mv) [38]. However, the Ch-Zn complex had a positive charge (+26.6) [46]. The low surface charge of the prepared nanoparticles (Ch-CuO and Ch-ZnO) may be due to the crosslinking reaction that blocked the hydroxyl and amino functional groups. The glutaraldehyde blocks the amino groups of chitosan while the hydroxyl groups were blocked by epichlorohydrin [29, 47, 48].

#### 3.2.5. FT-IR

**Figure 4:**
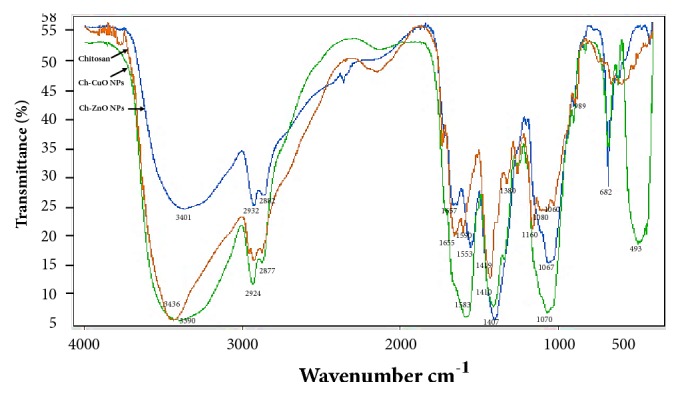
FT-IR spectra of chitosan (A), chitosan-copper oxide nanoparticles (Ch-CuO NPs), and chitosan-zinc oxide nanoparticles (Ch-ZnO NPs).

The FT-IR spectra of chitosan and Ch-MO NPs are shown in Figure 4. The spectrum of pure chitosan exhibits bands at 3436 cm^−1^ due to the stretching vibration mode of –OH and -NH_2_ groups. The peak at 2924 cm^−1^ is a type of C-H stretching vibration, while the band at 1655 cm^−1^ is due to the amide I group (C-O stretching along with N-H deformation mode). A band at 1590 cm^−1^ is attributed to the NH_2_ group due to N-H deformation, while a band at 1419 cm^−1^ is due to C-N axial deformation (amine group band). In addition, the peak at 1380 cm^−1^ peak is due to the COO^−^ group in carboxylic acid salt, and the band at 1160 cm^−1^ is assigned to the special broad peak of *β* (1–4) glucosidic bond in polysaccharide unit. However, the peak at 1080 cm^−1^ is attributed to the stretching vibration mode of the hydroxyl group, 989-1060 cm^−1^ stretching vibrations of C-O-C in glucose units [20].

The FT-IR spectrum of Ch-ZnO NPs exhibits band at 3401 cm^−1^ due to the combination between -OH and -NH_2_ groups. The peak at 2932 cm^−1^ is a typical of C-H stretch vibration. The band at 1657 cm^−1^ is due to the rest of amide I group while a band at 1553 cm^−1^ is attributed to the NH_2_ group due to N-H deformation. The peak at 1407 cm^−1^ is due to C-N axial deformation (amine group band). In addition, the band at 1067 cm^−1^ is attributed to the stretching vibration mode of the hydroxyl group and the band at 682 cm^−1^ ascribed to the vibration of O-Zn-O core groups.

The spectrum of Ch-CuO NPs exhibits bands at 3390 cm^−1^ due to the combination between -OH and -NH_2_ groups. The peak at 2924 cm^−1^ indicates a C-H stretching vibration. A band at 1583 cm^−1^ is attributed to the NH_2_ group due to N-H deformation, and 1410 cm^−1^ peak is due to C-N axial deformation (amine group band). A band at 1380 cm^−1^ is due to the COO- group in carboxylic acid salt, while the peak at 1070 cm^−1^ is attributed to the stretching vibration mode of the hydroxyl group. The band at 682 cm^−1^ is attributed to the vibration of O-Cu-O core groups. However, the peak at 493 is ascribed to Cu-O bond vibration.

In comparison with chitosan, the broader and stronger peak shifted considerably to lower wave number at 3390 cm^−1^ in Ch-CuO NPs and 3401 cm^−1^ in Ch-ZnO NPs, which indicates strong attachment of metal oxide to the amide groups of chitosan molecules (Figure 4). The absorption peaks at 2877-2924 in Ch-MO NPs are due to asymmetric stretching of CH_2_ and CH_3_ of chitosan polymer and the overlapping with -NH. The absorption peaks at 1583 and 1070 cm^−1^ in the spectrum of Ch-CuO NPs are attributed to bending vibration of the -NH group and the C-O stretching group but these peaks were observed at 1553 and 1067 cm^−1^ in spectrum of Ch-ZnO NPs. New broad absorption bands at 682 and 400 cm^−1^ were found in the FT-IR spectra of Ch-MO NPs which were ascribed to the vibration of O-Cu-O and O-Zn-O groups [49, 50].

### 3.3. Kinetic Studies of Adsorption Efficiency of Pesticides by Ch-MO NPs

**Figure 5:**
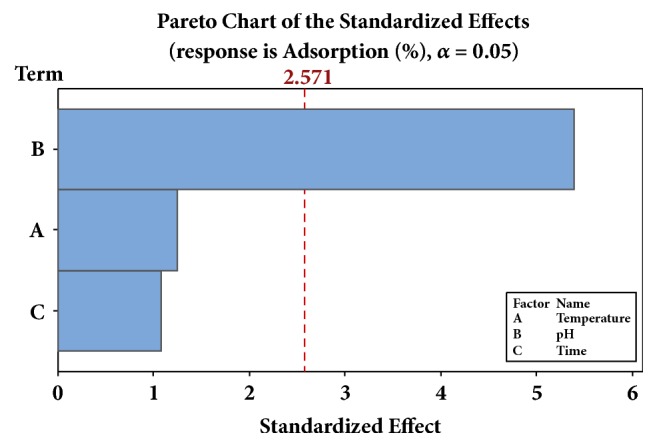
Pareto Chart of the standardized effects of pH, temperature, and time of adsorption (response is adsorption (%), *α* = 0.05).

Three factors (pH, temperature, and agitation time) were studied on the efficiency of Ch-CuO NPs in the adsorption of imidacloprid insecticide at 25 mg/L. The full factorial design was used in terms of the experimental runs, and the experimental data are shown in Table 2. The results indicate that the pH values of 7 and 9 showed the most significant effect on the adsorption efficiency of imidacloprid with 62.21, 84.24, 92.91, 100, and 87.43 for run 3, 4, 7, 8, and 9, respectively. To investigate the main effect of all factors, the adsorption efficiency was studied using the Pareto chart and the result is shown in Figure 5. The most affecting factor is the pH followed by temperature and then agitation time. The Pareto chart provides a clear visualization of the factor effects and indicates that the pH has the most significant effect on the adsorption at *α* = 0.05; however, the temperature and agitation time did not show values lower than the reference line (2.571 at *α* = 0.05) [2, 25]. From this analysis, the adsorption (%) can be calculated or predicted according to the following model (1).

(1) Adsorption  %=−73.3+0.479  Temperature+15.51  pH+0.413  TimeS=16.28  andR2=86.40%

It can be noted that the three factors have a positive sign that means that the adsorption will be increased with an increase in each factor. The factor has a greater correlation factor denoting the great effects. Therefore, the pH has a great effect (coefficient = 15.51) on the adsorption followed in the descending order by temperature (coefficient = 0.479) and then the agitation time (coefficient = 0.413). In addition, three-dimensional response surface plots are presented in Figure S4. These plots provide useful information about the behavior of the system within the experimental design, which was used to understand the main and interactive effects of the factors. The effect of pH, temperature, and agitation time on pesticides adsorption percentage was shown at initial concentration in Figure S4 right. The results indicated that the adsorption or retention percentage increased with increasing of the pH and temperature, but the optimum adsorption percentage was observed at pH 7 and temperature of 25°C. These results are consistent with the previous study, which reported that the removal rate of pyrethrin increased by an increase of pH to 8 [25]. The adsorption ratio increased at pH increase and induction time from 10 to 40 min, but the optimal adsorption was performed at pH 7 and after 25 minutes. However, the effect of time and temperature has proved the previous theory that confirmed that optimal temperature and induction time are from 25°C to 40°C and 25 to 40 minutes, respectively at the top of the surface plot curve. The contour plots shown in Figure S4 indicate the interaction between the pH and temperature and confirmed that the optimum adsorption was found at pH ranging from 6.5 to 9 with the optimal temperature from 25 to 40°C.

### 3.4. SPE of Pesticides Using Ch-MO NPs and HPLC Analysis

HPLC analytical methods for the tested pesticides were validated by calculating regression equation, correlation coefficient (R^2^), peak asymmetry factor (A_s_), LOD, and LOQ for each pesticide and the data are presented in Table 3. The values of R^2^ obtained for the regression lines demonstrate the excellent relationship between peak area and the injected amount of all pesticides (R^2^ ≥ 0.999). The LOD of the pesticides determined by HPLC ranged from 0.002 to 0.046 *μ*g/mL and the LOQ was in the range of 0.006 to 0.154 *μ*g/mL. The asymmetry factor (A_s_) is an indication for the peak tailing [51, 52] being in the range of 0.870 to 1.070.

The efficacy data of Ch-MO NPs (250 mg) in extraction and removal of pesticides from water samples at three levels (10, 50, and 100 mg/L) is presented in Tables 4 and 5 for Ch-CuO NPs and Ch-ZnO NPs, respectively, and compared to the standard ODS cartridge (Supelco) (Table 6). The data are presented as a percentage of that extracted by methanol: acetonitrile (50:50) and that found in water phase. It can be noted that the removal percentages were decreased with the increase of the concentration.  Table 4 shows the results of cartridge loaded with Ch-CuO NPs. All pesticides were adsorbed into the Ch-CuO NPs with high percentages compared to the amount remaining in the water phase. Lambda-cyhalothrin was the highest in removal (98.93, 95.19, and 92.66% at 10, 50, and 100 mg/L, respectively) followed in the descending order by abamectin (98.02, 94.34, and 92.31% at 10, 50, and 100 mg/L, respectively). However, there is no significant difference between both insecticides. Fenamiphos showed 95.33, 93.28, and 90.44% and then imidacloprid with 93.78, 90.39, and 72.91% at 10, 50, and 100 mg/L, respectively. However, methomyl and thiophanate-methyl showed moderate values (63.85-84.75%). Diazinon was the lowest pesticide among all the tested pesticides in removal percentages (70.15, 34.21, and 21.44% at 10, 50, and 100 mg/L, respectively). Ch-CuO NPs demonstrated that no amount of lambda-cyhalothrin was found in water at any of the tested concentrations. This finding may be due to the fact that the lambda-cyhalothrin has a very low solubility in water and a highest octanol-water partition coefficient value compared to the other tested pesticides [53], followed in the descending order by imidacloprid, thiophanate-methyl, fenamiphos, and abamectin. However, methomyl indicated high percentages in water (20.55, 25.00, and 33.37% at 10, 50, and 100 mg/L, respectively). This is may be due to the high solubility of this compound in the water [54].

All pesticides were also adsorbed into the Ch-ZnO NPs with high percentage compared to that found in the water phase and lambda-cyhalothrin was the highest in removal with 99.09, 98.00, 94.47% at 10, 50, and 100 mg/L, respectively (Table 5), followed in the descending order by abamectin, fenamiphos, and imidacloprid. However, diazinon and thiophanate-methyl showed moderate values (60.10-94.28%). Methomyl was the lowest pesticide among all tested pesticides (41.40, 38.51, and 36.62% at 10, 50, and 100 mg/L, respectively). These particles proved that the insecticide lambda-cyhalothrin was not detected in water at any of the tested concentrations. However, methomyl showed high percentages in water (18.09, 57.82, and 62.59% at 10, 50, and 100 mg/L, respectively).

Table 6 shows the recovery of pesticides at 10, 50, and 100 mg/L from water using the standard SPE cartridge (C_18_) obtained from Supelco. Diazinon, fenamiphos, and thiophanate-methyl were the most pesticides extracted from this type of cartridge in all tested concentrations. However, methomyl is still less compared to others. It can be observed that the standard SPE cartridge (C_18_) showed a disparity in extraction efficiency and was the least cartridge compared with Ch-CuO NPs and Ch-ZnO NPs in the recovery of most tested pesticides including abamectin (recovery of 48.11-97.59%), fenamiphos (recovery of 78.60-84.20%), imidacloprid (recovery of 31.20-80.16%), lambda-cyhalothrin (recovery of 51.70-93.88%), and methomyl (recovery of 23.35-40.37%). Unfortunately, the SPE has certain limitations, primarily related to low recovery, i.e., slightly lower sensitivity, in cases where the SPE column is blocked (blocking the absorption centers by the sample's solid and organic components) [55].

The enrichment factor (EF) of the prepared and standard cartridges is shown in Table 7. EF can be defined as the concentration of the analyte in organic phase to the original concentration in the aqueous phase. The results showed that the EF of Ch-CuO NPs ranged from 3.24 for diazinon to 8.73 for abamectin. However, there is no significant difference among the other pesticides. The EF of Ch-ZnO NPs ranged from 3.53 for methomyl to 8.83 for lambda-cyhalothrin. It can be noted that the EF values of the prepared cartridges were higher than the standard ODS (C_18_), which had a range of 2.91-7.43.

SPE became one of the most widely used treatment methods for various samples [56, 57]. This technology has many advantages, including high enrichment factor, easy operation, high recovery, rapid phase separation, low cost, low consumption of organic solvents, and effective matrix interference [58]. In the SPE process, the synthesis of adsorbents is the fundamental issue since the type and amount of absorbance largely determine selectivity, sensitivity, and full recovery. In general, properties with large surface areas, active surface locations, and a short propagation path can provide a significant number of improvements in extraction kinetics [59]. Compared with conventional adsorbents, nanoscale metal oxides have attracted more interest from researchers in recent years, given their high surface area and rapid absorption kinetics. Several results confirmed that the Ch-MO NPs were high adsorbent materials and used in SPE technique for extraction and removal of different pollutants [24, 25]. Ch-Zn was prepared and applied for removal of permethrin at optimum conditions, including adsorbent dose, agitating time, the initial concentration of pesticide, and pH on the adsorption [25]. The results indicated that the weight of 0.5 g of the bionanocomposite, at room temperature and pH 7, removed 99% of permethrin solution (25 mL, 0.1 mg L) using UV spectrophotometer at 272 nm. Copper-coated chitosan nanocomposite (Ch-Cu) was found to have high adsorption efficiency for parathion and methyl parathion, and maximum adsorption capacity of parathion was found to be 322.60 mg/g at an optimum pH of 2.0 [24]. This could be attributed to the inherent alkalinity of the adsorbent. In addition, high adsorption value of malathion could be explained by acidic hydrolysis of malathion to dithiophosphate followed by complexation of copper to form Cu (II) dithiophosphate. Ch-AgO NPs composite beads were also optimized to remove maximum permethrin as the model pesticide, with the amount of sorbent, agitating time, initial concentration of pesticide, and pH parameters [2]. In optimum conditions, room temperature and pH 7, the Ch-AgO NPs beads recovered 99% of permethrin solution (0.10 mg/L) using UV spectrophotometer compared to 50% with the pure chitosan.

### 3.5. Adsorption Isotherm Study

Adsorption isotherm models are important to determine the efficiency of the adsorption process. Adsorption isotherms illustrate the connection between the amount of adsorbed component per adsorbent weight and the concentration of the contaminated components in the solution. Determination of the adsorption parameters provides useful information, which can improve the adsorption efficiency of the systems. In the present study, the adsorption percentages were applied in Freundlich (1) and Langmuir (3) isotherm models as follows to predict which model is fit.

(2) q=KfC1/n

(3) q=qmax⁡KlC1+KlC

where q is adsorption capacity (*μ*g/g); K_f_ is Freundlich isotherm constant (*μ*g/g); C is concentration of the analyte (adsorbate) in the solution at equilibrium (*μ*g/mL), n is adsorption intensity; q_max⁡_ is maximum adsorption monolayer capacity (*μ*g/g); and K_l_ is Langmuir isotherm constant (mL/*μ*g).

By analyzing the linear correlation coefficient (R^2^) obtained, it is possible to identify the isotherm model that best represents the experimental data of this study [60]. From the values of R^2^ obtained (Table S2) for the Ch-MO NPs, it is possible to conclude that both of Langmuir and Freundlich isotherms are fit to this study with R^2^ > 0.92. When the experimental data follows the Langmuir model, this assumes that a monomolecular layer is formed when adsorption takes place without any interaction between the adsorbed molecules. However, the data follows the Freundlich isotherm, which means that the adsorption process takes place on heterogeneous surfaces and adsorption capacity is related to the concentration of the analyte at equilibrium [61]. The maximum adsorption capacity (q_max⁡_) of Ch-MO NPs was observed for all the tested pesticides. The Ch-CuO NPs and Ch-ZnO NPs showed the highest adsorption capacities (2.50 × 10^4^ and 1.00 × 10^5^ *μ*g/g, respectively) for thiophanate-methyl compared to 1.00 × 10^4^ *μ*g/g by using ODS (C_18_). However, the insecticide methomyl showed a low *q*_max⁡_ on Ch-CuO NPs and Ch-ZnO NPs (2.00 × 10^3^, 1.00 × 10^3^ *μ*g/g, respectively) compared to 2.86 × 10^2^ by using ODS (C_18_).

## 4. Conclusion

Novel Ch-MO NPs, stationary phases for SPE technique, were prepared and characterized by FT-IR, SEM, TEM, XRD, and Zeta-Nano-sizer. The chromatographic retention behaviors of seven pesticides on Ch-MO NPs were investigated and compared with standard ODS (C_18_ column). The factors of the pH, temperature and agitation time were studied on the efficiency of these products in adsorption or retention of imidacloprid insecticide and the results proved that the pH was the most significant factor. It was reported that the Ch-MO NPs are able to remove the selected pesticides at the optimum condition of agitation time 25 min, pH 7, and 25°C. Ch-CuO NPs and Ch-ZnO NPs exhibited high selectivity for the tested pesticides as solutes and the extracted amount by these products was more than the ODS in most cases at three levels of concentrations (10, 50 and 100 mg/L in aqueous solution). The new adsorbent nanoparticles behaved as a reversed phase retention mechanism based on hydrophobic interaction as well as inclusion interactions and weak hydrophilicity for the polar pesticides such as methomyl based on partitioning and surface adsorption process. The nanoparticles will possess great prospect in chromatographic analysis especially SPE and SPME techniques. In addition, these products are newly biocompatible, environmentally friendly, and low cost to extract and clean-up pesticides from wastewater. In future, this work will be conducted on the packing of the HPLC columns with these products as new alternatives to the current stationary phases for separation of pesticide residues.

## Figures and Tables

**Table 1:** Reaction conditions and characterizations of chitosan-metal oxide nanoparticles (Ch-MO NPs).

**Product code **	**Reaction components**	**Mole ratio**	**Product color**	**Yield (**%**) **	**Particles diameter (nm) ± SE**	**Zeta-potential (mV)**
Ch-CuO NPs	Chitosan: Cu_2_O: Glutaraldehyde:	1:2:2:3	Yellowish-dark	85.29	93.74±5.70	+0.516
	Epichlorohydrin					
Ch-ZnO NPs	Chitosan: ZnO: Glutaraldehyde:	1:4:2:3	Yellowish	91.67	97.95±9.46	+0.086
	Epichlorohydrin					

**Table 2:** Experimental design using Minitab software and standardized effects of temperature, pH, and time on the adsorption of imidacloprid insecticide at 25 mg/L on Ch-CuO NPs.

**Run order**	**Temperature (**°**C)**	**pH**	**Time (min)**	**Adsorption (**%**) ± SE**
1	10	5	10	12.18±0.58
2	40	5	10	31.86±1.16
3	10	9	10	62.21±0.62
4	40	9	10	84.24±0.78
5	10	5	40	19.23±1.77
6	40	5	40	27.93±2.01
7	10	9	40	92.91±1.72
8	40	9	40	100.00±0.00
9	25	7	25	87.43±0.98

**Table 3:** Statistical data from regression analysis of different pesticides obtained from the study with analytical HPLC methods.

**Pesticide**	**Rt (min) ± SD**	**A**_**s**_ ** ± SD**	**Regression equation**	**R** ^**2**^	LOD (*μ*g/mL)	LOQ (*μ*g/mL)
Abamectin	7.999 ± 0.01	0.871 ± 0.00	y = 4523.45190x-2.70225	0.9998	0.023	0.077
Diazinon	7.975 ± 0.00	0.870 ± 0.01	y = 1177.60010x+0.42100	0.9999	0.046	0.154
Fenamiphos	3.374 ± 0.01	0.885 ± 0.01	y = 3214.11453x+0.89949	0.9997	0.002	0.006
Imidacloprid	3.647 ± 0.00	0.853 ± 0.04	y = 4728.25710x+0.794634	0.9998	0.020	0.066
Lambda-cyhalothrin	10.761 ± 0.05	0.923 ± 0.05	y = 2874.16095x+0.431849	0.9999	0.012	0.040
Methomyl	2.795 ± 0.03	0.953 ± 0.00	y = 4972.13330x+3.61685	0.9997	0.018	0.059
Thiophanate-methyl	4.566 ± 0.01	1.070 ± 0.00	y = 3412.34475x+11.24269	0.9997	0.024	0.081

**Rt**: retention time. **A**_**s**_: peak asymmetry factor. **R**^**2**^: linear correlation coefficient. **LOD**: limit of detection. **LOQ**: limit of quantification.

**Table 4:** Efficiency of Ch-CuO NPs in adsorption of different pesticides using solid phase extraction cartridge technique.

**Pesticides**	**Removal (**%**) **±** SE at three levels of concentration (mg/L)**			**Active ingredient (**%**) found in water **±** SE**			**Total found (**%**)**^**∗**^ ± **SE**		
	**(Extracted in methanol / acetonitrile)**			**(Remaining in water sample)**					
	**10**	**50**	**100**	**10**	**50**	**100**	**10**	**50**	**100**
Abamectin	98.02^a^ ± 3.41	94.74^a^ ± 1.02	92.31^a^ ± 0.23	0.00^f^ ± 0.00	2.53^e^ ± 0.47	3.94^e^ ± 0.09	98.02^a^ ± 4.55	97.27^a^ ± 1.35	96.25^a^ ± 0.13
Diazinon	70.15^b^ ± 1.46	34.21^e^ ± 1.10	21.44^f^ ± 0.25	18.89^b^ ± 1.06	19.05^b^ ± 1.03	23.81^b^ ± 0.60	89.04^a^ ± 2.01	53.27^c^ ± 1.98	45.25^c^ ± 0.72
Fenamiphos	95.33^a^ ± 1.69	93.28^ab^ ± 0.99	90.44^a^ ± 1.04	4.03^d^ ± 0.18	4.67^d^ ± 0.15	7.31^d^ ± 0.09	99.36^a^ ± 1.59	97.94^a^ ± 0.63	97.76^a^ ± 0.32
Imidacloprid	93.78^a^ ± 0.45	90.39^b^ ± 0.61	72.91^d^ ± 0.30	5.80^c^ ± 0.28	8.16^c^ ± 0.09	25.96^b^ ± 0.96	99.58^a^ ± 0.22	99.75^a^ ± 0.49	98.87^a^ ± 0.58
Lambda-cyhalothrin	98.44^a^ ± 1.01	95.14^a^ ± 0.41	92.66^a^ ± 0.07	0.00^f^ ± 0.00	0.00^f^ ± 0.00	0.00^f^ ± 0.00	98.44^a^ ± 0.58	95.14^a^ ± 0.14	92.66^b^ ± 0.02
Methomyl	77.15^b^ ± 0.28	70.17^d^ ± 0.00	63.85^e^ ± 0.39	20.55^a^ ± 0.27	25.00^a^ ± 0.65	33.37^a^ ± 2.34	97.70^a^ ± 0.48	95.16^a^ ± 0.57	97.22^a^ ± 0.79
Thiophanate-methyl	84.75^b^ ± 1.82	78.91^c^ ± 0.89	74.62^c^ ± 0.22	5.14^e^ ± 0.11	8.19^c^ ± 0.04	22.47^c^ ± 0.10	89.89^a^ ± 0.92	87.10^b^ ± 0.86	97.09^a^ ± 0.16

*∗* The values lower than 100% mean the nonextracted amount of pesticide from Ch-CuO NPs. Values are mean of three replicates and are given as mean ± standard error. Different letters in the same column indicate significant differences according to Student-Newman-Keuls (SNK) test (P≤0.05).

**Table 5:** Efficiency of Ch-ZnO NPs in adsorption of different pesticides using solid phase extraction cartridge technique.

**Pesticides**	**Removal (**%**) ± SE at three levels of concentration (mg/L)**			**Active ingredient (**%**) found in water ± SE **			**Total found (**%**)**^**∗**^ ± **SE**		
	**(Extracted in methanol / acetonitrile)**			**(Remaining in water sample)**					
	**10**	**50**	**100**	**10**	**50**	**100**	**10**	**50**	**100**
Abamectin	98.72^a^ ± 5.31	93.15^a^ ± 0.65	92.63^a^ ± 0.66	0.00^e^ ± 0.00	1.84^e^ ± 0.18	2.51^e^ ± 0.06	98.72^a^ ± 4.05	94.99^b^ ± 0.26	95.15^b^ ± 0.57
Diazinon	94.28^b^ ± 1.53	76.12^b^ ± 1.14	72.55^c^ ± 1.22	5.25^b^ ± 0.47	18.08^b^ ± 0.25	23.01^b^ ± 0.36	99.54^a^ ± 1.72	94.15^b^ ± 0.42	95.56^b^ ± 0.89
Fenamiphos	95.21^b^ ± 3.53	93.33^a^ ± 0.92	87.20^b^ ± 0.44	4.34^c^ ± 0.36	4.42^d^ ± 0.13	7.52^de^ ± 0.04	99.55^a^ ± 2.79	97.75^ab^ ± 0.68	94.72^b^ ± 0.40
Imidacloprid	96.90^ab^ ± 0.35	97.76^a^ ± 0.68	88.47^b^ ± 0.61	1.69^d^ ± 0.16	2.21^e^ ± 0.16	10.42^cd^ ± 0.07	99.58^a^ ± 0.22	99.97^a^ ± 0.76	98.88^a^ ± 0.27
Lambda-cyhalothrin	99.09^a^ ± 0.78	98.00^a^ ± 1.61	94.47^c^ ± 0.41	0.00^e^ ± 0.00	0.00^f^ ± 0.00	0.00^f^ ± 0.00	99.09^a^ ± 0.55	98.00^ab^ ± 1.00	94.47^b^ ± 0.20
Methomyl	41.47^d^ ± 1.08	38.51^c^ ± 0.31	36.62^f^ ± 0.56	18.09^a^ ± 0.27	57.82^a^ ± 0.25	62.59^a^ ± 0.33	59.56^b^ ± 1.18	96.33^ab^ ± 0.09	99.21^a^ ± 0.78
Thiophanate-methyl	90.62^c^ ± 0.86	60.60^b^ ± 0.52	60.10^e^ ± 0.22	3.49^c^ ± 0.04	6.66^c^ ± 0.14	12.34^c^ ± 0.07	94.11^a^ ± 0.88	67.26c ± 0.38	72.44^c^ ± 0.18

*∗* The values lower than 100% mean the nonextracted amount of pesticide from Ch-ZnO NPs. Values are mean of three replicates and are given as mean ± standard error. Different letters in the same column indicate significant differences according to Student-Newman-Keuls (SNK) test (P≤0.05).

**Table 6:** Efficiency of standard ODS cartridge (Supelco) in adsorption of different pesticides using SPE technique.

**Pesticides**	**Removal efficiency (**%**) ± SE at three levels of concentration (mg/L)**			**Active ingredient found in water (**%**) ± SE **			**Total found (**%**)**^**∗**^ ± **SE**		
	**(Extracted in methanol / acetonitrile)**			**(Remaining in water sample)**					
	**10**	**50**	**100**	**10**	**50**	**100**	**10**	**50**	**100**
Abamectin	97.59^a^ ± 2.51	95.4^a^4 ± 0.48	48.11^c^ ± 0.17	00.00^e^ ± .00	4.28^e^ ± 0.43	11.86^d^ ± 0.65	97.59^a^ ± 2.51	99.72^a^ ± 0.45	59.97^c^ ± 0.34
Diazinon	99.36^a^ ± 2.05	96.28^a^ ± 0.43	87.65^a^ ± 0.28	00.00^e^ ± 0.00	2.00^f^ ± 0.04	7.45^e^ ± 0.67	99.36^a^ ± 2.05	98.32^a^ ± 0.42	95.10^a^ ± 0.47
Fenamiphos	84.20^b^ ± 3.04	78.28^b^ ± 0.46	78.60^b^ ± 0.41	14.45^a^ ± 0.65	16.54^b^ ± 0.29	16.96^c^ ± 0.25	98.65^a^ ± 1.84	94.82^a^ ± 0.56	95.56^a^ ± 0.45
Imidacloprid	80.16^b^ ± 1.03	51.26^c^ ± 0.45	31.20^d^ ± 1.19	8.11 ± ^d^0.11	13.90^c^ ± 0.14	36.84^a^ ± 0.23	88.27^a^ ± 0.98	65.16^c^ ± 0.34	68.04^b^ ± 0.71
Lambda-cyhalothrin	93.88^a^ ± 1.21	72.05^b^ ± 2.46	51.70^9c^ ± 0.55	00.00^e^ ± .00	7.42^d^ ± 0.34	10.64^d^ ± 0.65	93.88^a^ ± 1.21	79.47^b^ ± 1.49	62.43^b^ ± 0.60
Methomyl	40.37^d^ ± 0.63	28.20^d^ ± 0.46	23.35^d^ ± 1.08	11.87^c^ ± 0.87	13.99^c^ ± 0.87	22.98^b^ ± 0.98	52.24^b^ ± 0.76	42.19^d^ ± 0.63	46.33^d^ ± 0.96
Thiophanate-methyl	78.98^c^ ± 4.26	75.30^b^ ± 0.40	74.28^b^ ± 0.22	13.07^b^ ± 0.00	19.65^a^ ± 0.65	24.67^b^ ± 0.83	92.05^a^ ± 4.26	94.95^a^ ± 0.53	98.95^a^ ± 0.52

*∗* The values lower than 100% mean the nonextracted amount of pesticide from standard solid phase extraction cartridge. Values are mean of three replicates and are given as mean ± standard error. Different letters in the same column indicate significant differences according to Student-Newman-Keuls (SNK) test (P≤0.05).

**Table 7:** Enrichment factor (EF) of Ch-Si MNPs for adsorption of different pesticides from water sample.

**Pesticides **	**EF ± SE of Ch-MO NPs at three levels of pesticide concentrations (** ***μ*** **g/mL)**											
	**10**	**50**	**100**	**Mean ± SE**	**10**	**50**	**100**	**Mean ± SE**	**10**	**50**	**100**	**Mean ± SE**
	**Ch-CuO NPs**				**Ch-ZnO NPs**				**ODS (Supelco)**			
Abamectin	8.22	9.47	8.51	8.73^a^ ± 0.31	8.28	9.31	8.54	8.71^a^ ± 0.26	8.19	9.58	4.43	7.40^a^ ± 1.26
Diazinon	5.24	2.74	1.75	3.24^b^ ± 0.85	7.04	6.10	5.93	6.36^ab^ ± 0.28	7.42	7.71	7.17	7.43^a^ ± 0.13
Fenamiphos	7.56	7.35	7.24	7.38^a^ ± 0.08	7.55	7.35	6.98	7.29^ab^ ± 0.14	6.68	6.16	6.28	6.37^b^ ± 0.13
Imidacloprid	7.39	7.60	5.12	6.70^ab^ ± 0.65	7.64	8.22	6.22	7.36^ab^ ± 0.49	6.32	4.59	2.19	4.37^c^ ± 0.98
Lambda-cyhalothrin	7.87	10.80	7.31	8.66^a^ ± 0.89	7.93	11.13	7.45	8.83^a^ ± 0.95	7.37	8.18	4.08	6.54^b^ ± 1.03
Methomyl	9.34	5.64	4.31	6.43^ab^ ± 1.24	5.02	3.10	2.47	3.53^c^ ± 0.63	4.89	2.27	1.58	2.91^d^ ± 0.83
Thiophanate-methyl	6.76	6.32	5.97	6.35^ab^0.19	7.23	4.85	4.81	5.63^bc^ ± 0.66	6.30	6.03	5.94	6.09^b^ ± 0.09

Values are mean of three replicates and are given as mean ± standard error. Different letters in the same column indicate significant differences according to Student-Newman-Keuls (SNK) test (P≤0.05).

## Data Availability

All data generated or analyzed during this study are included in this article. In addition, the related datasets are available from the corresponding author on reasonable request.

## Supplementary Materials

Supplementary Materials Figure S1 shows the chemical structures of tested pesticides (abamectin, chlorpyrifos methyl, diazinon, fenamiphos, imidacloprid, lambda-cyhalothrin, methomyl, and thiophanate-methyl). Figure S2 shows the 3D-schematic diagram for preparation mechanism of Ch-MO NPs. Figure S3 shows the zeta potential distribution graph of Ch-MO NPs. Figure S4 presents the surface plot and contour plot of the adsorption (%) of imidacloprid insecticide on Ch-CuO NPs versus temperature, pH, and agitation time. Table S1 shows a summary of the methods conditions used for determination of different pesticides by HPLC system. Table S2 indicates the parameters of the isothermal models of Ch-MO NPs for adsorption of different pesticides.
