# Supplementary material for: Development of a Solid-Phase Extraction (SPE) Cartridge Based on Chitosan-Metal Oxide Nanoparticles (Ch-MO NPs) for Extraction of Pesticides from Water and Determination by HPLC
Source: Int J Anal Chem. 2018 Oct 2;2018:3640691. doi: 10.1155/2018/3640691 (PMC6189673; doi:10.1155/2018/3640691)
Supplement: Supplementary Materials — Figure S1 shows the chemical structures of tested pesticides (abamectin, chlorpyrifos methyl, diazinon, fenamiphos, imidacloprid, lambda-cyhalothrin, methomyl, and thiophanate-methyl). Figure S2 shows the 3D-schematic diagram for preparation mechanism of Ch-MO NPs. Figure S3 shows the zeta potential distribution graph of Ch-MO NPs. Figure S4 presents the surface plot and contour plot of the adsorption (%) of imidacloprid insecticide on Ch-CuO NPs versus temperature, pH, and agitation time. Table S1 shows a summary of the methods conditions used for determination of different pesticides by HPLC system. Table S2 indicates the parameters of the isothermal models of Ch-MO NPs for adsorption of different pesticides. [file 3640691.f1.pdf]

SUPPLEMENTAL MATERIAL

Development of a solid-phase extraction (SPE) cartridge based on chitosan-metal oxide nanoparticles (Ch-MO NPs) for extraction of pesticides from water and determination by HPLC

Mohamed E. I. Badawy*, Mahmoud A. M. El-Nouby, Abd El-Salam M. Marei

Department of Pesticide Chemistry and Technology, Faculty of Agriculture, 21545-El-Shatby, Alexandria University, Alexandria, Egypt

* Address correspondence to Mohamed E. I. Badawy, Department of Pesticide Chemistry and Technology, Faculty of Agriculture, 21545-El-Shatby, Alexandria University, Alexandria, Egypt. Phone: 002039575269; Fax: 002035972780; E-mail: m_eltaher@yahoo.com

3.5. Adsorption isotherm study

Adsorption isotherm models are important to determine the efficiency of the adsorption process. Adsorption isotherms illustrate the connection between the amount of adsorbed component per adsorbent weight and the concentration of the contaminated components in the solution. Determination of the adsorption parameters provides useful information, which can improve the adsorption efficiency of the systems. In the present study, the adsorption percentages were applied in Freundlich (1) and Langmuir (2) isotherm models as follows to predict which model is fit.

$$q=K_f C^{\frac{1}{n}} \ldots\ldots\ldots\ldots\ldots\ldots\ldots (1)$$

$$q=\frac{q_{max} K_l C}{1+K_l C} \ldots\ldots\ldots\ldots\ldots\ldots\ldots (2)$$

Where q = adsorption capacity (μg/g); K_f = Freudlich isotherm constant (μg/g); C = concentration of the analyte (adsorbate) in the solution at equilibrium (μg/mL), n = adsorption intensity; q_{max} = maximum adsorption monolayer capacity (μg/g); K_l = Langmuir isotherm constant (mL/μg).

By analyzing the linear correlation coefficient (R^2) obtained, it is possible to identify the isotherm model that best represent the experimental data of this study [1]. From the values of R^2 obtained (Table S2) for the Ch-MO NPs, it is possible to conclude that both of Langmuir and Freundlich isotherms are fit to this study with $R^2 > 0.92$. When the experimental data follows the Langmuir model, this assumes that a monomolecular layer is formed when adsorption takes place without any interaction between the adsorbed molecules. However, the data follows the Freundlich isotherm, this means that the adsorption process takes place on heterogeneous surfaces and adsorption capacity is related to the concentration of the analyte at equilibrium [2]. The maximum adsorption capacity (q_{max}) of Ch-MO NPs was observed for all the tested pesticides. The Ch-CuO

NPs and Ch-ZnO NPs showed the highest adsorption capacities (2.50×10^4 and 1.00×10^5 μg/g, respectively for thiophanate-methyl compared to 1.00×10^4 μg/g by using ODS (C_{18}). However, the insecticide methomyl showed a low q_{max} on Ch-CuO NPs and Ch-ZnO NPs (2.00×10^3, 1.00×10^3μg/g, respectively) compared to 2.86×10^2 by using ODS (C_{18}).

References

1. P. Silva, J. Francisco, J. Cajé, R. Cassella, and W. Pacheco. "A batch and fixed bed column study for fluorescein removal using chitosan modified by epichlorohydrin," *Journal of Environmental Science and Health, Part A,* vol. 53, pp. 55-64, 2018.

2. F. Naseeruteen, N. S. A. Hamid, F. B. M. Suah, W. S. W. Ngah, and F. S. Mehamod. "Adsorption of malachite green from aqueous solution by using novel chitosan ionic liquid beads," *International Journal of Biological Macromolecules,* vol. 107, pp. 1270-1277, 2017.

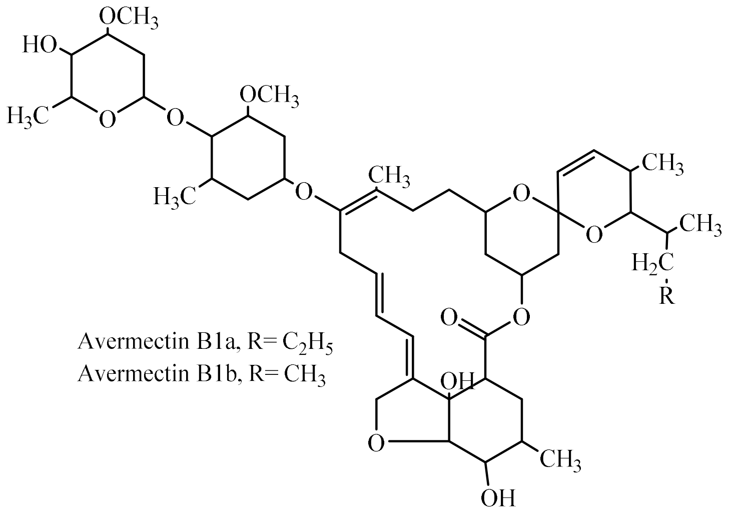

Abamectin

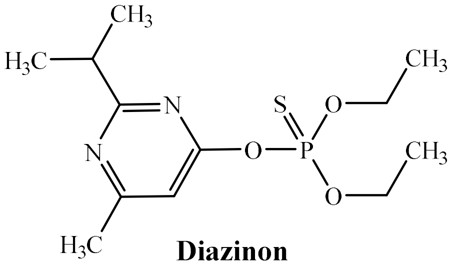

Diazinon

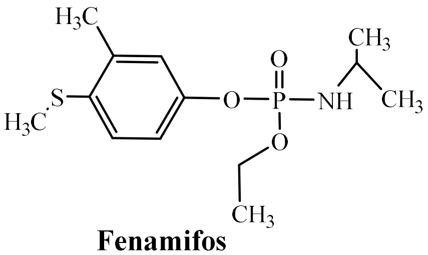

Fenamifos

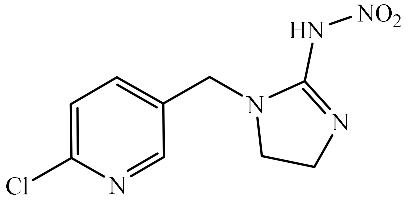

Imidacloprid

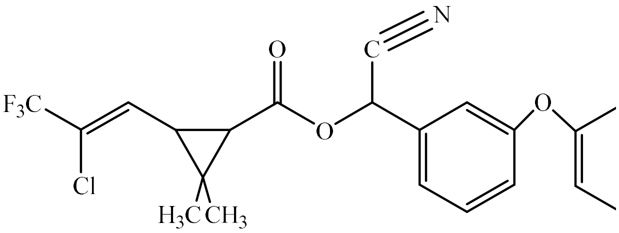

Lambda-cyhalothrin

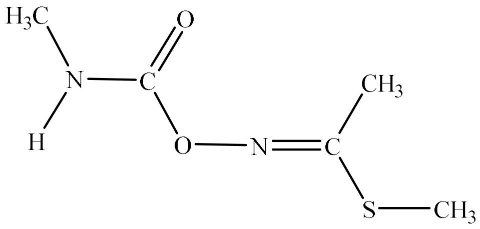

Methomyl

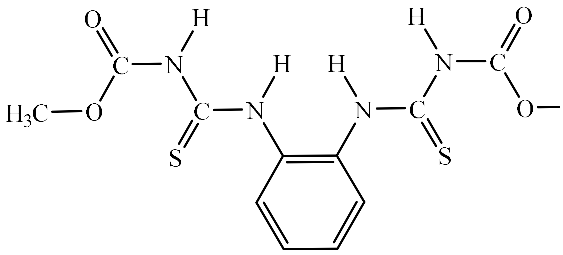

Thiophanate-methyl

Figure S1. Abamectin is the International Organization for Standardization (ISO)-approved common name for a mixture of the components avermectin B1a (≥ 80%) and avermectin B1b (≤ 20%). Chlorpyriphos methyl, diazinon and fenamifos are organophosphorus insecticides. Imidacloprid is a neonicotinoid insecticide. Lambda-cyhalothrin is a pyrethroid insecticide. Methomyl is a crbamate insecticide and thiophanate-methyl is a thiocarbamate fungicide.

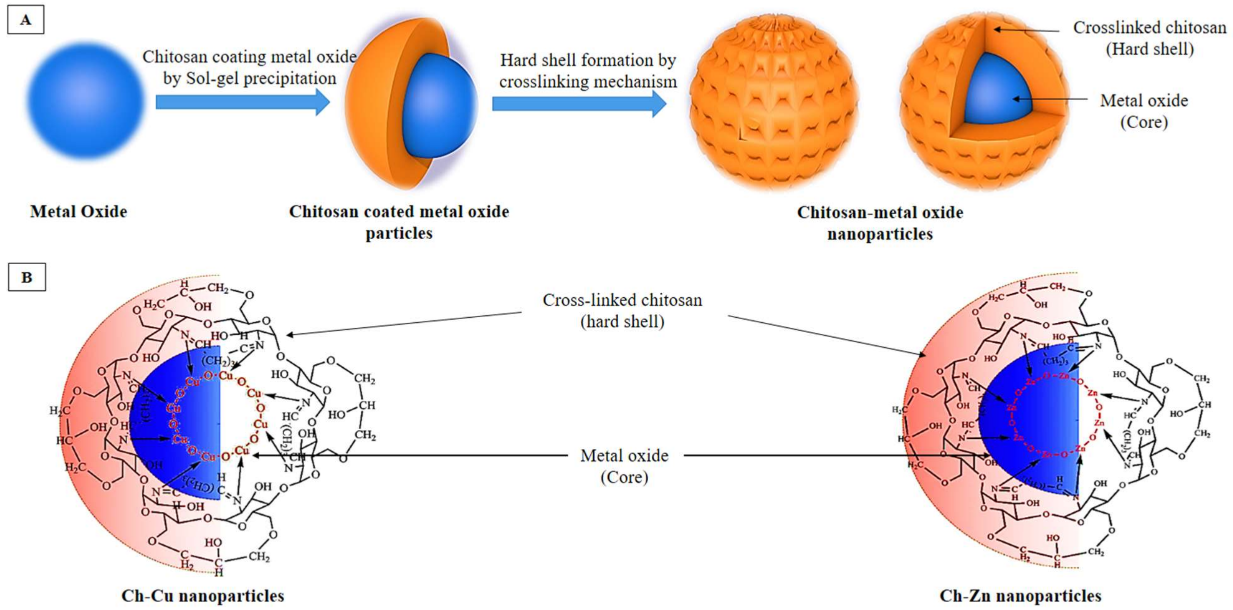

Figure S2. 3D-schematic diagram for preparation mechanism of chitosan-metal oxide nanoparticles (Ch-MO NPs) (**A**) and proposed chemical structure (**B**).

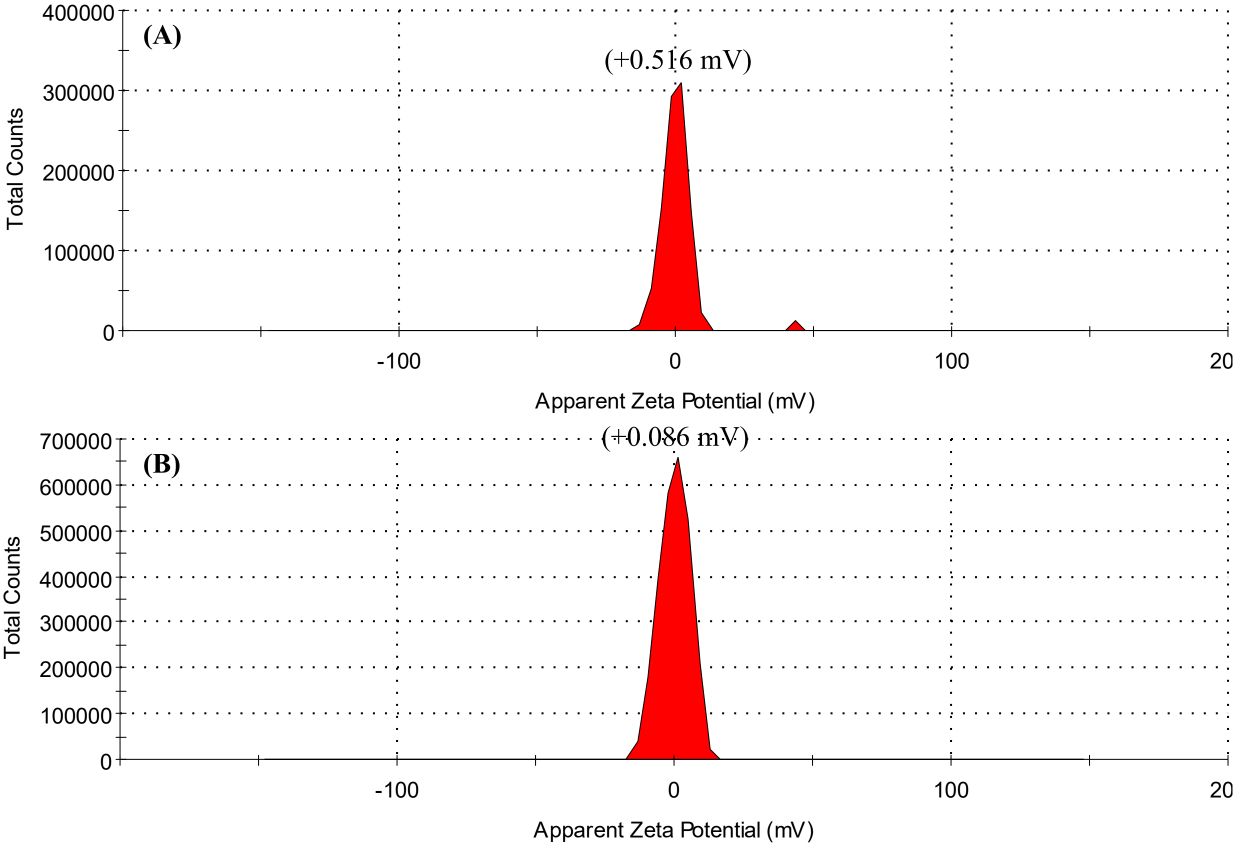

Figure S3. Zeta potential distribution graph of chitosan-metal oxide nanoparticles (Ch-MO NPs) (A) chitosan-copper oxide nanoparticles (Ch-CuO NPs) and (B) chitosan-Zinc oxide nanoparticles (Ch-ZnO NPs).

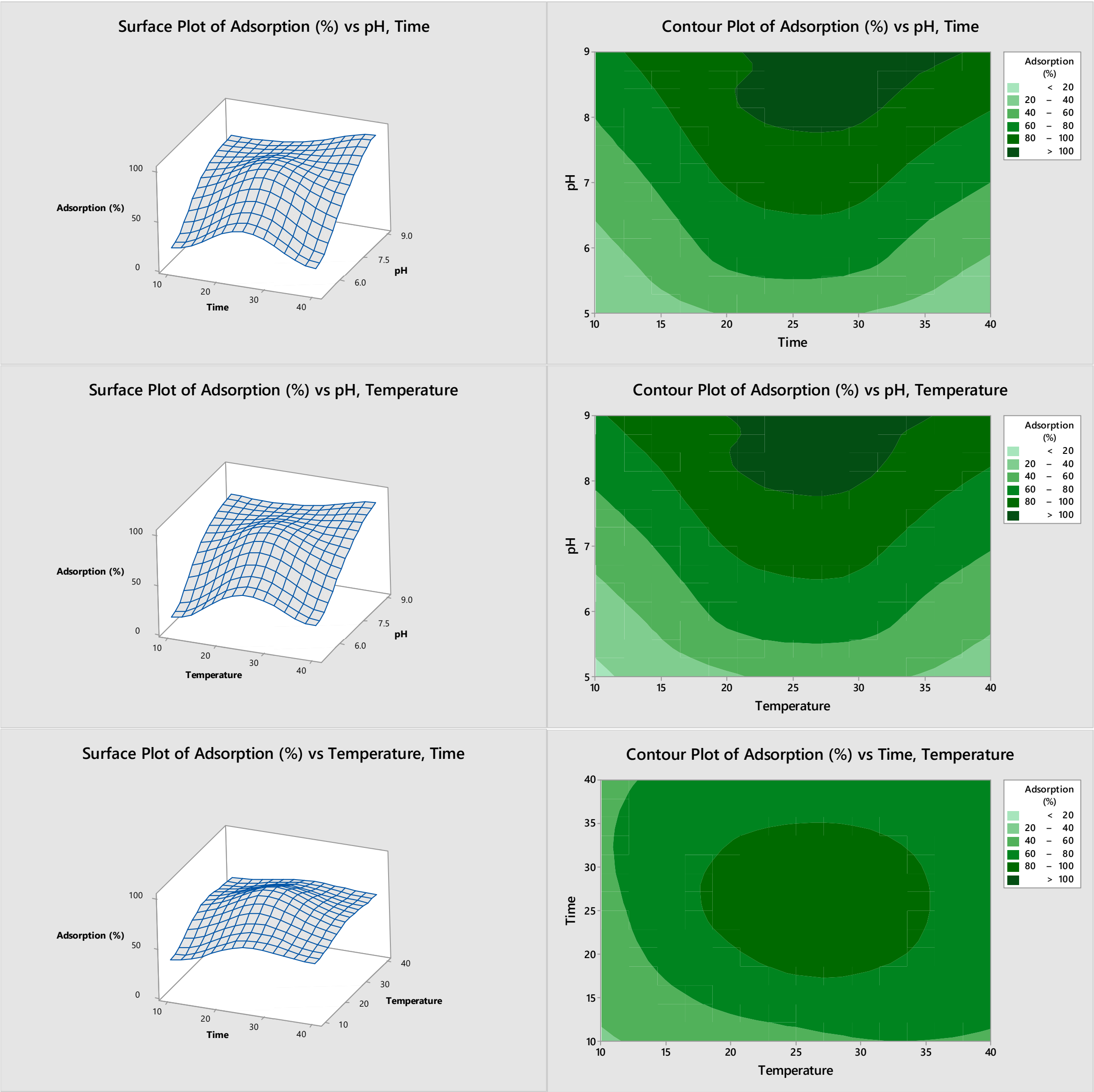

Figure S4. Surface plot and contour plot of the adsorption (%) of imidacloprid insecticide on Ch-CuO NPs vs temperature, pH and agitation time.

Table S1. Summary of methods conditions used for determination of different pesticides by HPLC system

Pesticide	Column	Chromatographic conditions				
		**Mobile phase composition (% by volume)**	**Flow rate (mL/min)**	**Column temperature (°C)**	**Detector wavelength (nm)**	**Elution system**
Abamectin	ZORBAX Eclips Plus C18	Acetonitrile:Methanol:Water (10:80:10)	1.0	40	245	Isocratic
Diazinon	ZORBAX Eclips Plus C18	Methanol:Water (40:60)	1.0	25	252	Isocratic
Fenamiphos	ZORBAX Eclips Plus C18	Acetonitrile:Water (40:60)	1.0	40	249	Isocratic
Imidacloprid	ZORBAX Eclips Plus C18	Acetonitrile:Water (40:60)	1.0	40	269	Isocratic
Lambda-cyhalothrin	ZORBAX Eclips Plus C18	Acetonitrile:Water (80:20)	1.0	40	289	Isocratic
Methomyl	ZORBAX Eclips Plus C18	Acetonitrile:Water (60:40)	1.0	30	233	Isocratic
Thiophanate-methyl	ZORBAX Eclips Plus C18	Acetonitrile:Methanol:Water (20:30: 50)	1.0	30	269	Isocratic

Table S2. Parameters of the isothermal models of Ch-MO NPs for adsorption of different pesticides

Pesticides	Sorbents	Freundlich			Langmuir		
		R^2	K_f (μg/g)	n	R^2	q_{max} (μg/g)	K_l (mL/μg)
Abamectin	Ch-CuO NPs	0.99	-0.50	1.02	0.99	2.50×10^{3}	-1.48×10^{-3}
	Ch-ZnO NPs	0.99	-0.48	1.02	0.99	3.33×10^{3}	-1.14×10^{-3}
	ODS (C_{18})	0.92	-0.12	1.27	0.98	5.00×10^{3}	7.31×10^{-4}
Diazinon	Ch-CuO NPs	0.99	-0.07	1.93	0.99	2.22×10^{2}	1.64×10^{-2}
	Ch-ZnO NPs	0.99	-0.33	1.13	0.99	1.67×10^{3}	2.35×10^{-3}
	ODS (C_{18})	0.99	-0.41	1.05	0.99	1.00×10^{4}	4.01×10^{-4}
Fenamiphos	Ch-CuO NPs	0.99	-0.51	1.01	0.99	1.67×10^{4}	2.30×10^{-4}
	Ch-ZnO NPs	0.99	-0.46	1.03	0.99	1.00×10^{4}	3.83×10^{-4}
	ODS (C_{18})	0.99	-0.57	1.03	0.99	5.00×10^{3}	6.80×10^{-4}
Imidacloprid	Ch-CuO NPs	0.99	-0.35	1.10	0.99	3.33×10^{3}	1.15×10^{-3}
	Ch-ZnO NPs	0.99	-0.46	1.03	0.99	1.43×10^{4}	2.73×10^{-4}
	ODS (C_{18})	0.95	0.03	1.64	0.99	3.70×10^{2}	1.04×10^{-2}
Lambda-cyhalothrin	Ch-CuO NPs	0.98	-0.70	0.59	0.99	3.33×10^{3}	-1.15×10^{-3}
	Ch-ZnO NPs	0.98	-0.66	0.94	0.99	3.33×10^{3}	-1.17×10^{-3}
	ODS (C_{18})	0.99	-0.37	1.10	0.99	2.00×10^{3}	1.95×10^{-3}
Methomyl	Ch-CuO NPs	0.99	-0.43	1.12	0.99	2.00×10^{3}	1.29×10^{-3}
	Ch-ZnO NPs	0.99	-1.07	1.10	0.99	1.00×10^{3}	1.75×10^{-3}
	ODS (C_{18})	0.99	-0.39	1.46	0.99	2.86×10^{2}	6.83×10^{-3}
Thiophanate-methyl	Ch-CuO NPs	0.99	-0.78	1.00	0.99	2.50×10^{4}	1.17×10^{-4}
	Ch-ZnO NPs	0.99	-0.96	1.00	0.99	1.00×10^{5}	2.41×10^{-5}
	ODS (C_{18})	0.99	-0.63	1.03	0.99	1.00×10^{4}	3.17×10^{-4}

K_f: Freundlich constant indicate the degree of adsorption, **n**: adsorption intensity, and **K_l**: Langmuir constant indicate the force of adsorption. **q_{max}**: maximum adsorption monolayer capacity.
